# Phenotypic characterization and resilience assessment of wheat genotypes under single and combined abiotic stresses

**DOI:** 10.3389/fpls.2026.1954462

**Published:** 2026-10-15

**Authors:** Lei Sun, Liya Niu, Xueping Shi, Bo Jiao, Chunhong Ma, Fan Yang, Fushuang Dong, Shuo Zhou

**Affiliations:** 1 Hebei Key Laboratory of Plant Genetic Engineering, Institute of Biotechnology and Food Science, Hebei Academy of Agriculture and Forestry Sciences, Shijiazhuang, China; 2 Institute of Crop Genetics and Breeding, Cangzhou Academy of Agriculture and Forestry Sciences, Cangzhou, China; 3 Hebei Science and Technology Innovation Service Center, Shijiazhuang, China

**Keywords:** combined stress, nitrogen deficit, resilience, water deficit, wheat

## Abstract

**Background:**

Climate change and limited resources are intensifying abiotic stresses in wheat production, creating an urgent need for varieties that combine high yield with reliable performance under variable conditions. However, selecting for yield under optimal or single-stress conditions often fails to predict how genotypes will perform under complex, real-world challenges.

**Methods:**

We evaluated 56 wheat genotypes under four environments: fully fertilized and irrigated (FFI), water deficit (WD), nitrogen deficit (ND), and combined water-nitrogen deficit (CWND). To quantify resilience, we developed a composite D-value index by integrating stress-response ratios of seven agronomic traits using PCA, fuzzy membership normalization, and entropy-weighted aggregation.

**Results:**

Both WD and ND reduced grain yield by 21–22% relative to FFI, while CWND caused a further decline (–31%), representing the greatest reduction among all treatments. Stress environments fundamentally altered the relationships between key agronomic traits. Genotype performance varied considerably across environments: XM296 maintained relatively high yield under single stresses but showed limited multi-trait stability under combined stress; LX99 showed stable performance across diverse stresses while retaining high yield potential; in contrast, XN20 exhibited relatively high tolerance at the expense of low productivity.

**Conclusions:**

Given the environment-dependent nature of resilience, breeding strategies should balance broad adaptation with improvements targeted at the dominant stress patterns in key production regions. This approach will better translate genetic gains into stable yields in farmers’ fields.

## 1 Introduction

Agricultural production worldwide is predominantly limited by the availability of nitrogen (N) and water (Mueller et al., 2012). Climate change further exacerbates these pressures by altering soil carbon dynamics and water availability (Qiu et al., 2023). Wheat, which accounts for over 40% of the global human diet, stands out as a major crop with notably low nitrogen use efficiency (NUE), with only 30–35% of applied N fertilizer being absorbed (Raun and Johnson, 1999; Gaju et al., 2011; Das et al., 2026). Nitrogen fertilizers account for approximately 60% of global annual fertilizer consumption (Wang et al., 2018). Current worldwide demand exceeds 100 million metric tons per year, and projections suggest it could rise by an additional 200 million metric tons by 2050 to meet the growing demands of global food production (Mirzaee and Nafchi, 2025). Similarly, escalating water scarcity challenges in many regions have heightened the need for efficient water use (Liu et al., 2022; Wei et al., 2024).

The critical role of both nitrogen and water in agricultural productivity cannot be overstated. However, the increased reliance on these resources in agriculture has led to rising environmental and economic costs (Lassaletta et al., 2013). The pollutant effects of mineral N fertilizers (Galloway et al., 2008) and water scarcity pose significant challenges for sustainable agricultural practices. Consequently, there is an urgent need to explore genotypes that maximize the efficiency of both N and water use.

To address these challenges, numerous strategies have been developed to improve nitrogen and water use efficiency. These include optimized fertilizer scheduling, improved irrigation practices, and sensor-based monitoring (Arregui and Quemada, 2008; Pancorbo et al., 2021). While these approaches offer promising pathways to mitigate resource waste and environmental impacts, they often face practical limitations in real-world farming systems. For instance, optimizing irrigation can alleviate water deficit, while precision N management can reduce fertilizer overuse (Morison et al., 2008). However, these methods are not always sufficient to ensure stable yields under varying environmental conditions.

An alternative and complementary route is to identify or breed crop varieties that perform well under low nitrogen or limited water conditions. Such resilient genotypes can maintain stable yields with fewer inputs, offering a sustainable way to cope with resource scarcity and climate variability (Blum and Jordan, 1985). Growing drought-resistant wheat varieties, for example, forms the foundation for increasing yield and water productivity (Morison et al., 2008). Integrating better genetics with smarter management is therefore key to building more efficient and adaptive agricultural systems.

Drought or nutrient stress tolerance is typically defined as how well a genotype performs relative to others under the same stress conditions (Hall et al., 1993). Because this definition focuses on relative performance, genotypes should be evaluated in paired environments: one under stress and one under non-stressed conditions. A common and effective way to do this is by expressing key agronomic traits as ratios of their values under stress versus optimal conditions (Fernandez, 1992). These ratio indices help account for differences in inherent yield potential among genotypes and reduce the influence of field variability, making them more reliable indicators of true stress resilience.

Despite advances in high-throughput phenotyping, wheat breeding under stress still relies heavily on grain yield or yield-related traits (Ivić et al., 2021; Sallam et al., 2024). Yet directly selecting for yield under nitrogen deficiency, water deficit, or combined stress remains difficult. Yield is a highly polygenic trait, and its heritability drops sharply under stress, mainly because the intensity of stress varies across space and time in field trials (Blum, 2018). To complement yield-based approaches, secondary traits such plant height, tiller number, spikelet number, grains per spike, and thousand-grain weight are widely used for screening, as they respond sensitively to resource limitations and correlate with stress adaptation (Lopes et al., 2012; Zhang et al., 2016; Ahmad et al., 2020; Kadkol et al., 2020). In parallel, physiological and biochemical indicators including chlorophyll content, hormones such as gibberellic acid (GA3) and abscisic acid (ABA) accumulation, and antioxidant enzyme activity, have also proven valuable for assessing stress responses (Song et al., 2017; Ahmed et al., 2019; Lamlom et al., 2025). Furthermore, identifying specific stress-responsive proteins (e.g., TaHSP17.4) and their underlying genetic basis provides potential molecular targets for selection (Wang et al., 2023). Many of these traits can be assessed early in development, facilitating rapid phenotyping and shortening breeding cycles (Roychowdhury et al., 2024).

Given the polygenic and multidimensional nature of stress tolerance, composite indices that integrate multiple correlated traits offer a more holistic assessment of resilience. Such indices may provide a more comprehensive reflection of the underlying genetic architecture and have shown potential to enhance breeding efficiency compared to single-trait selection in several studies (Pour-Aboughadareh et al., 2025). However, the diversity of stress-responsive traits complicates their integration in large-scale evaluations. Principal component analysis (PCA) is frequently employed to reduce dimensionality and assign data-driven weights to individual traits (Ahmed et al., 2020; Bhandari et al., 2024; Rezk et al., 2025). Complementary methods including membership function analysis, cluster analysis, correlation analysis, and grey relational analysis further enhance the reliability of multi-trait assessments under adverse conditions (Lai et al., 2009; Sun et al., 2021). For example, the membership function value of drought tolerance (MFVD), calculated from multiple physiological and agronomic indicators, enables quantitative ranking of genotypes according to their integrated stress response (Yan et al., 2009).

While numerous studies have ranked wheat genotypes for performance under either water or nitrogen limitation (Zhao et al., 2012; Memon et al., 2022; Darwish et al., 2023; Raya-Sereno et al., 2024), systematic comparisons that include both single and combined deficits alongside optimal conditions remain rare. Yet, as climate variability and input constraints increasingly expose crops to multiple concurrent stresses, breeding programs require reliable strategies to identify genotypes that maintain stability. In this study, we assessed wheat genotypes under four controlled conditions: fully fertilized and irrigated (FFI), water deficit (WD), nitrogen deficit (ND), and combined water–nitrogen deficit (CWND). Rather than relying solely on grain yield, we integrated stress-to-control ratios of multiple agronomic traits into a composite resilience index. This index was constructed using principal component analysis and an entropy weight method to objectively assign component weights, improving upon conventional eigenvector-based approaches. Our strategy enables simultaneous identification of high-yielding and broadly resilient genotypes for targeted breeding under resource-limited environments.

## 2 Materials and methods

### 2.1 Field experimental conditions and environmental stress treatments

The field experiment was conducted from October 2021 to June 2022 at the Dahe Experimental Station, Institute of Biotechnology and Food Science, Hebei Academy of Agricultural and Forestry Sciences, Shijiazhuang, China. Four environmental treatments were established to represent contrasting resource availability scenarios: fully fertilized and irrigated (FFI); water deficit (WD), nitrogen deficit (ND), and combined water and nitrogen deficit (CWND). In the FFI treatment, nitrogen was applied in two splits: 140 kg N ha^-^¹ as basal fertilizer at sowing and 100 kg N ha^-^¹ as topdressing at stem elongation: along with 105 kg P_2_O_5_ ha^-^¹ and 120 kg K_2_O ha^-^¹ applied at sowing. Irrigation totaling 150 mm was delivered in two equal applications (75 mm each) at stem elongation and anthesis. The WD treatment received identical fertilization as FFI but no supplemental irrigation, relying solely on natural rainfall. In contrast, the ND treatment received no nitrogen fertilizer while maintaining the same P and K rates and receiving the standard 150 mm of irrigation. The CWND treatment combined both stresses, thus representing a rainfed, low-input production scenario. Each treatment was implemented in a separate large field block to avoid cross-contamination of water and nutrient regimes.

### 2.2 Experimental design and field layout

The experiment was established in a single field characterized by uniform soil texture and baseline fertility. The entire experimental field was systematically partitioned into four contiguous management zones. Each zone was exclusively assigned to one of the four water–nitrogen regimes: fully fertilized and irrigated (FFI), water deficit (WD), nitrogen deficit (ND), and combined water- and nitrogen-deficit (CWND). Within each of the four environmental zones, the 56 genotypes were arranged in three complete blocks, with genotypes randomized within each block. Each genotype subplot consisted of five 1.0-meter-long rows, spaced 25 cm apart. Seeds were sown at a within-row spacing of 6.7 cm. The two outer rows of each subplot served as guard rows to reduce edge effects. From each plot, five plants were sampled at harvest. Standard agronomic practices such as pest and weed control in the region were uniformly applied across the entire field, with the strict exception of the targeted water and nitrogen interventions specific to each zone.

### 2.3 Phenotypic trait measurement and data collection

Seven agronomic traits were measured to evaluate phenotypic performance under four contrasting water and nitrogen supply conditions. At maturity, five plants were randomly selected from the inner rows of each plot to minimize border effects, and used for measurement of plant height (PH), spike length (SL), and tiller number (TN). PH was recorded as the distance from the base to the tip of the main spike (excluding awns). SL was measured along the rachis of the main spike, and TN was counted as the number of fertile tillers per plant. The Aboveground dry weight (AGDW) was air-dried to constant weight. Grain number per spike (GN30) was recorded by counting all grains from the threshed main spikes of the five sampled plants. Thousand kernel weight (TKW) was analyzed using an automated seed testing system and thousand grain weight analysis system (Model SC-G; WSeen, Hangzhou, China), which provided high-throughput measurement of individual grain weight based on image analysis. Grain yield (GY) was determined by harvesting the entire plot using a small-scale combine harvester, followed by threshing and weighing the total grain mass. Phenotypic values represent measurements from a single plot per genotype per treatment, with five plants sampled for morphological and yield component traits. All measurements were performed under consistent environmental conditions at harvest.

To investigate the temporal dynamics of wheat canopy chlorophyll status in response to nitrogen and water availability, SPAD values were measured on the flag leaves of 72 genotypes (56 described above and 16 newly included; **Supplementary Table S1**) across four environmental treatments. These 16 additional genotypes were assessed for SPAD only and did not contribute to the analysis of other agronomic traits. Measurements were taken at three key phenological stages: stem elongation, heading, and grain filling, using a portable chlorophyll meter (SPAD-502, Konica Minolta). For each genotype within a plot, SPAD readings were averaged across three positions (base, middle, tip) of the flag leaf from five randomly selected plants.

### 2.4 Statistical analysis

Statistical analyses were conducted using a combination of frequentist and Bayesian approaches to comprehensively assess the relationships between agronomic traits and grain yield across contrasting water–nitrogen regimes. All statistical analyses were conducted in R (v4.3.1). To avoid pseudoreplication, plot-level means (averaged across the five sampled plants per plot) were used as the observational unit, and all downstream analyses were performed on the resulting genotype-by-environment mean matrix. The four water–nitrogen regimes (FFI, WD, ND, CWND), implemented in physically distinct field zones, were treated as separate environments​ within a multi-environment trial framework.

Treatment effects. A linear mixed model was fitted to the genotype-by-environment means using the lme4 package, with environment treated as a fixed effect and genotype as a random intercept:


$$Y_{ij}=\mu +E_{i}+G_{j}+\epsilon_{ij}$$

where $E_{i}$ represents the fixed water–nitrogen regime effect, $G_{j}∼N(0,\sigma_{G}^{2})$ accounts for genetic variation among genotypes, and $\epsilon_{ij}$ captures residual variance encompassing both genotype-by-environment interaction and experimental error. Significance of fixed effects was assessed via the *lmerTest* package (Kuznetsova et al., 2017). Estimated marginal means for treatments were computed using *emmeans (*Lenth, 2024), and pairwise comparisons were adjusted for multiple testing with the Tukey method. Significance letters were assigned using the compact letter display (CLD) method as implemented in the *multcomp* R package (Hothorn et al., 2008).

Genotype-by-environment interaction. G×E interaction for grain yield was characterized using AMMI and GGE biplot analyses based on genotype-by-environment means. In the AMMI analysis, the G×E interaction matrix was decomposed by singular value decomposition (SVD) after removing genotype and environment main effects. The proportion of variance explained by each interaction principal component axis (IPCA) was calculated, and an AMMI1 biplot was constructed using trait means and IPCA1 scores. For GGE analysis, SVD was applied to the environment-centered data matrix. The first two principal component scores were used to generate a which-won-where biplot, with mega-environments and winning genotypes identified via the convex hull polygon.

Temporal dynamics of SPAD values under different water-nitrogen regimes were analyzed using repeated-measures analysis of variance. The model included treatment and developmental period as fixed effects, with genotype included as a random effect to account for repeated measurements on the same plants over time. Analyses were performed using the afex package (Barr et al., 2013) in R (v4.3.1), which automatically applies appropriate corrections for sphericity violations when necessary. Estimated marginal means for all treatment-period combinations were computed using the emmeans package. Pairwise comparisons between treatments at each developmental period, as well as across developmental periods within each treatment, were conducted with Tukey’s adjustment for multiple testing. Significant differences (α = 0.05) were visualized using compact letter display. All analyses were performed on SPAD measurements collected at three developmental stages: stem elongation, heading, and grain filling.

To further explore the drivers of grain yield (GY) under varying resource conditions, a Bayesian multiple regression model was constructed using the brms package (Bürkner, 2017). The model regressed GY on six key agronomic predictors:


GY ~TKW + GN30 + TN + AGDW +SL +PH

The Bayesian model assumed a Gaussian likelihood and was fitted using Markov chain Monte Carlo (MCMC) sampling with four independent chains, each running for 2,000 iterations (1,000 warm-up draws discarded). Model fit was assessed via the Bayesian R², calculated using the r2_bayes() function from the performance package. This posterior-based metric measures the Bayesian model’s explanatory power for the response variable’s variability (Gelman et al., 2019). Complementing the Bayesian regression, correlation analysis was performed between each predictor and GY within each treatment group using the cor() function from base stats package.

### 2.5 Comprehensive drought and nutrient stress resilience evaluation using a PCA–entropy weighting system

For each of the seven measured agronomic traits, a stress response coefficient (DCj​) was first calculated for every genotype as the ratio of its trait value under stress to that under fully fertilized and irrigated (FFI) control conditions (Equation 1):

(1) $$DC_{ij}=\frac{X_{ij}^{stress}}{X_{ij}^{FFI}}$$

Where $X_{ij}^{stress}$ and $X_{ij}^{FFI}$ denote the observed value of trait *j* for genotype *i* under stress and optimal conditions, respectively. This ratio normalizes performance relative to each genotype’s own baseline, thereby isolating stress-specific responses from inherent genetic differences in trait magnitude. The resulting values $\;DC_{ij}$ were then standardized (Z-score transformed) and subjected to principal component analysis (PCA) separately for each stress environment. Only components with eigenvalues greater than 1 were retained, yielding three principal components (PCs) per stress treatment that collectively explained approximately 70% of the total multivariate variance. The score of genotype *i* on the *k*-th PC was computed as Equation 2:

(2) $$PC_{ik}=\;\sum_{j=1}^{m=7}\alpha_{jk}*Z_{ij}$$

Where $\alpha_{jk}$ is the loading of trait *j* on PC *k*, and is the standardized $DC_{ij}$.

To integrate these orthogonal components into a unified metric, each genotype’s PC score was transformed using a linear fuzzy membership function to a comparable 0–1 scale (Equation 3):

(3) $$U_{ik}=\;\frac{PC_{ik}-\text{min}(PC_{k})}{\max(PC_{k})-\text{min}(PC_{k})}$$

where $\;U_{ik}$ represents the normalized adaptive performance of genotype *i* along the biological dimension captured by PC *k*. The relative contribution of each PC to the final index was then determined objectively via the entropy weight method, which assigns higher weights to components exhibiting greater discriminative power across genotypes (Deng et al., 2023). The entropy weight was derived as Equation 4:

(4) $$\begin{array}{r} f_{ik}=\;\frac{U_{ik}}{\sum_{i=1}^{n}U_{ik}}\\ E_{k}=\;-\frac{1}{\ln n}\sum_{i=1}^{n}f_{ik}\text{ln}f_{ik}\\ w_{k}=\;\frac{1-E_{k}}{\sum_{k=1}^{m=3}\left(1-E_{k}\right)} \end{array}$$

Where $f_{ik}$ is the proportion of genotype ‘s membership value on PC; $E_{k}$ is the information entropy of PC; $\;w_{k}$ is the entropy-derived weight for PC; *n* is the total number of genotypes; *m* is the number of retained PCs.

Finally, a composite stress resilience index ( $D_{i}$) was calculated for each genotype as the weighted sum of its membership-transformed PC scores Equation 5:

(5) $$D_{i}=\sum_{k=1}^{m=3}w_{k}*U_{ik}$$

The resulting ranges from 0 to 1, with higher values indicating superior overall resilience across multiple physiological and agronomic dimensions. This integrated metric served as the foundation for comprehensive genotype assessment across stress environments.

## 3 Results

### 3.1 Phenotypic variation across environments

Phenotypic responses of wheat 56 genotypes across the four water-nitrogen treatments (FFI, WD, ND, CWND) revealed considerable variation in grain yield and six associated agronomic traits. To visualize how genotypes performed across these conditions, we generated radar plots for each trait, scaling values from 0 to 1 within each environment to allow comparison. The plots arranged treatments in concentric layers, with WD at the center, followed by ND, CWND, and FFI on the outermost ring. In terms of GY, JM22 performed best under FFI, D4366 under WD, and JM47 under ND. GY9415 exhibited the highest for grain number under WD and FFI, H0628 in ND while HG35 performed best under CWND. These patterns highlight clear differences among genotypes in how they respond to specific stresses, some excel under drought, others under low nitrogen, with no single genotype dominating across all conditions (**Figure 1**; **Supplementary Figure S1**).

**Figure 1:**
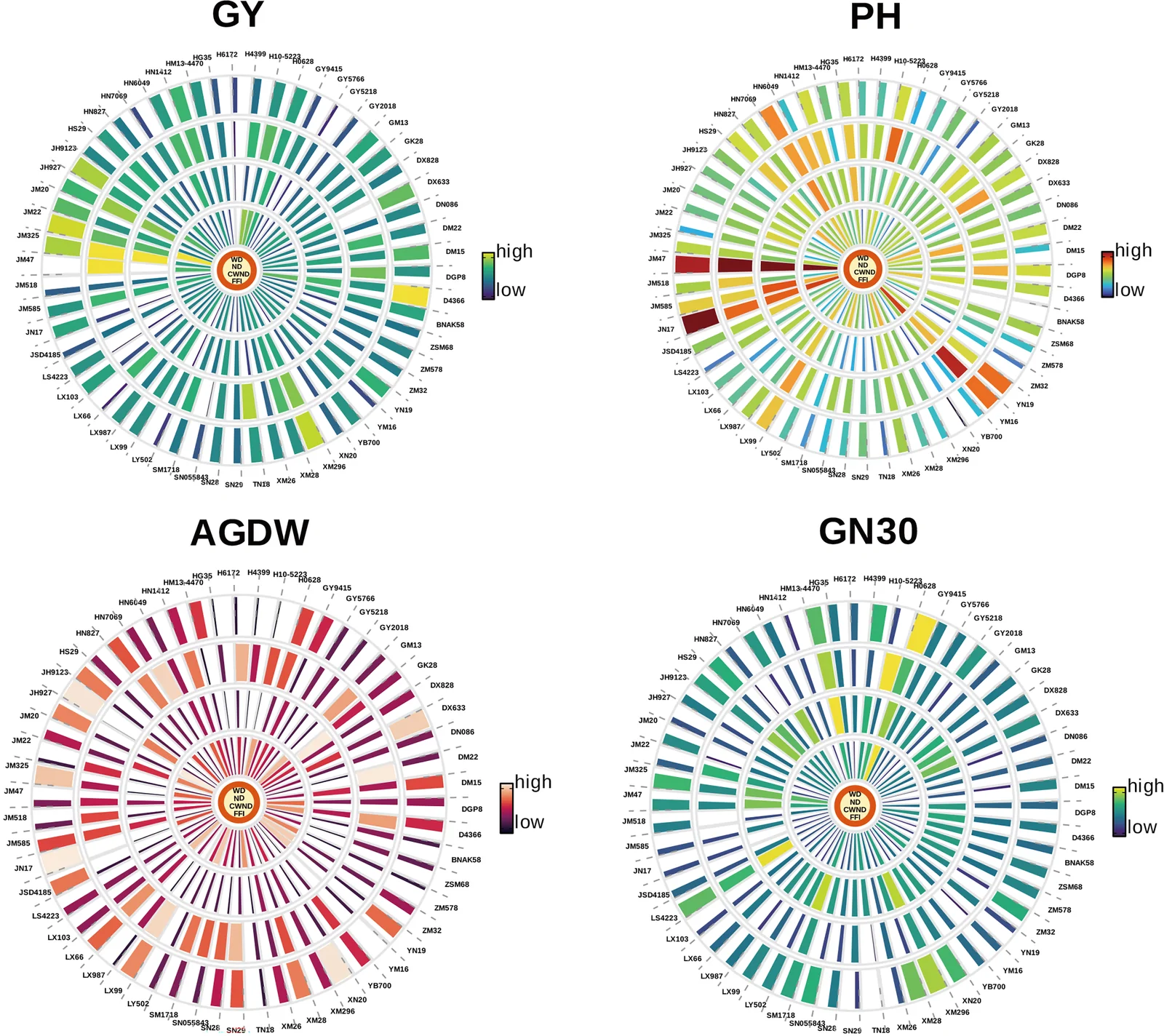
Phenotypic profiling of agronomic traits across four environmental treatments. Data from 56 wheat genotypes were collected under well-fertilized and irrigated (FFI), water deficit (WD), nitrogen deficit (ND), and combined water–nitrogen deficit (CWND) conditions. Trait values were normalized to a 0–1 scale within each environment and visualized using concentric radar plots, with WD at the innermost ring and FFI at the outermost.

Trait values were consistently highest in FFI and lowest in CWND, reflecting the cumulative impact of combined stress (**Supplementary Figure S2**). AGDW was significantly higher in FFI (49.51 ± 11.57 g) and ND (46.20 ± 10.77 g) than in WD (37.48 ± 7.73 g) and CWND (33.40 ± 6.28 g). GN30 was highest in FFI (1091.34 ± 180.16), intermediate in both WD (963.14 ± 161.79) and ND (988.23 ± 158.13), and lowest in CWND (855.95 ± 128.01). FFI differed significantly from all other treatments, and CWND was significantly lower than both WD and ND. GY was highest in FFI (0.77 ± 0.14 kg), lower in both WD (0.61 ± 0.11 kg) and ND (0.60 ± 0.09 kg), and lowest in CWND (0.53 ± 0.08 kg). FFI was significantly greater than all stress treatments, while CWND was significantly lower than both WD and ND; the latter two treatments did not differ significantly from one another. PH was highest in FFI (85.18 ± 5.31 cm) and ND (83.89 ± 5.10 cm), and lower in WD (78.72 ± 5.43 cm) and CWND (78.77 ± 4.88 cm). FFI and ND were not significantly different, nor were WD and CWND. In contrast, TKW showed no significant differences among treatments, consistent with its known resilience to moderate environmental variation (**Table 1**; **Figure 2**; **Supplementary Figure S3**).

**Table 1:** Summary statistics of agronomic traits across different environmental treatments.

Trait	FFI	WD	ND	CWND
AGDW (g)	49.51 ± 11.57 b	37.48 ± 7.73 a	46.20 ± 10.77 b	33.40 ± 6.28 a
GN30	1091.34 ± 180.16 c	963.14 ± 161.79 b	988.23 ± 158.13 b	855.95 ± 128.01 a
GY (kg)	0.77 ± 0.14 c	0.61 ± 0.11 b	0.60 ± 0.09 b	0.53 ± 0.08 a
PH (cm)	85.18 ± 5.31 b	78.72 ± 5.43 a	83.89 ± 5.10 b	78.77 ± 4.88 a
SL (cm)	8.96 ± 0.91 c	8.65 ± 0.67 b	8.70 ± 0.75 bc	8.26 ± 0.81 a
TKW (g)	46.13 ± 3.63 a	45.85 ± 4.54 a	45.70 ± 4.94 a	46.12 ± 3.33 a
TN	12.86 ± 2.62 c	11.42 ± 1.59 b	11.99 ± 2.69 bc	9.17 ± 0.85 a

**Figure 2:**
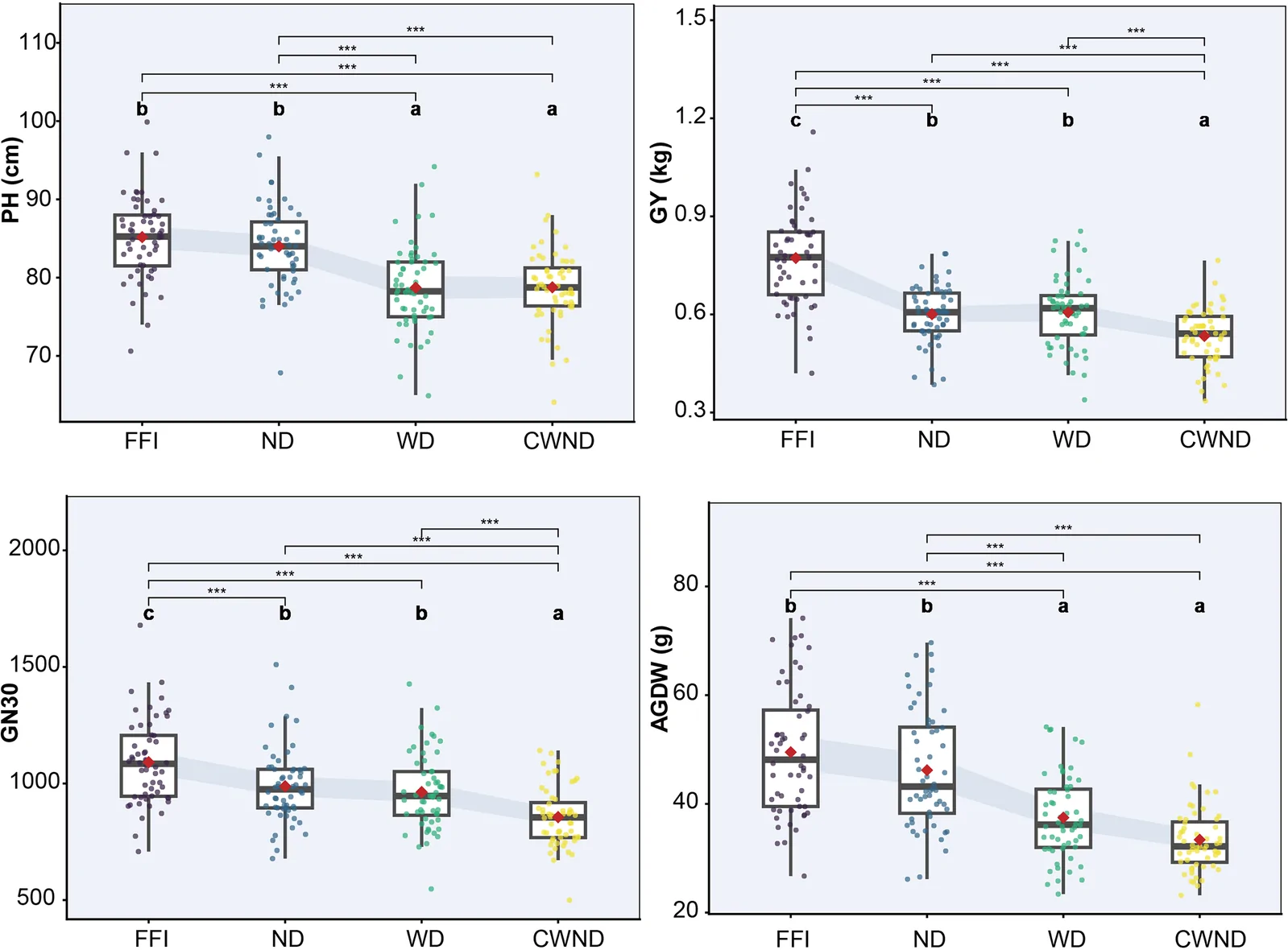
Distribution and statistical comparison of key agronomic traits across nitrogen–water deficit treatments. Boxplots show the performance of four traits—plant height (PH), grain number per 30 spikes (GN30), grain yield (GY), and aboveground dry weight (AGDW)—in 56 wheat genotypes under four treatments: fully fertilized and irrigated (FFI), water deficit (WD), nitrogen deficit (ND), and combined water–nitrogen deficit (CWND). Boxes represent interquartile ranges, horizontal lines indicate medians, red diamonds denote means, and individual points are raw observations. Significant differences between treatments were assessed using Tukey’s HSD test (**p < 0.001); letters (A, B, C) above boxes indicate homogeneous groups.

Variability also shifted across conditions (**Supplementary Table S2**). Most traits were most variable under FFI, for instance, AGDW (CV = 23.4%), GN30 (CV = 16.5%) and SL (CV = 10.2%). However, some became more variable under stress; for example, TN under ND had a coefficient of variation (CV) of 22.4%, compared to 20.4% in FFI, suggesting that genetic potential for most traits is more fully expressed when resources are abundant. Skewness and kurtosis values indicated that most trait distributions were close to normal, with only minor deviations under stress.

Together, these results showed that water and nitrogen deficits reduced yield and enhanced genotypic variation, as reflected in higher trait coefficients of variation under stress conditions compared to normal condition.

### 3.2 Environment-dependent reconfiguration of trait covariation patterns

Correlation analysis revealed that TKW and GN30 were positively associated under both nitrogen and water deficit conditions. AGDW showed a positive association with TN under nitrogen deficit, while PH and GY tended to co-vary under water deficit. Most other trait pairs exhibited weak or non-significant correlations, indicating limited coordination among agronomic traits across stress environments (**Supplementary Figure S4**; **Supplementary Table S3**).

Bayesian multivariate regression was used to estimate the effect of each agronomic trait on grain yield, adjusting for inter-trait correlations. This approach accounts for multicollinearity among predictors and provides probabilistic estimates of effect direction and magnitude. The models explained modest proportions of yield variance, with Bayesian values of 0.207 (95% CI [0.072, 0.344]) under WD, 0.149 ([0.042, 0.276]) under ND, and 0.130 ([0.034, 0.252]) under CWND.

Across all three stress environments, aboveground dry weight (AGDW) showed a positive association with grain yield, but the strength of evidence varied. Under WD, AGDW had a posterior mean of 0.322, a 95% HDI ranging from -0.083 to 0.715, and a direction probability (pd) of 93.9%, indicating a positive trend. Under CWND, AGDW also showed a positive mean (0.227) with a 95% HDI of [-0.0902, 0.546] and pd of 91.6%. In contrast, under ND, the posterior mean was 0.194, but the 95% HDI included zero ([-0.381, 0.762]), and pd was only 75.8%, suggesting a weak positive trend.

Other traits exhibited highly environment-specific patterns. Spike length (SL) was positively associated with yield only under WD (mean = 0.189, 95% HDI [-0.102, 0.481], pd = 89.5%). Under ND and CWND, SL showed no clear directional signal (HDI including zero; pd = 62.2% and 64.8%). Plant height (PH) showed a weak negative trend under WD (mean = -0.179, 95% HDI [-0.458, 0.0946], pd = 90.2%) and CWND (mean = -0.0235, 95% HDI [-0.329, 0.278], pd = 56.0%), but a weak positive trend under ND (mean = 0.226, 95% HDI [-0.0597, 0.511], pd = 94.0%). Grain number per 30 panicles (GN30) showed a tendency towards a negative effect under WD (mean = -0.147, 95% HDI [-0.449, 0.166], pd = 83.6%), but this did not reach the threshold for strong evidence. Under ND and CWND, GN30 showed no conclusive evidence (HDI including zero; pd = 74.0% and 53.1%). Notably, under dual stress (CWND), most traits showed reduced effect sizes and elevated uncertainty, with 95% HDIs often overlapping zero.

Principal component analysis (PCA) was conducted separately under four treatments. The first two principal components explained 46.5% (WD), 46.1% (ND), and 47.3% (CWND) of total phenotypic variance.

Under WD, PC1 was primarily loaded by AGDW and TN; GY showed a moderate positive loading on PC1. Under ND, PC1 was also dominated by AGDW and TN, while GY and GN30 loaded more strongly on PC2. Under CWND, PC1 was mainly driven by SL, followed by AGDW and PH; both GN30 and GY exhibited positive loadings on PC2.

These results showed although the overall phenotypic variation is similarly structured across environments, the functional meaning of the major axes of variation is environment-specific (**Figure 3B**).

**Figure 3:**
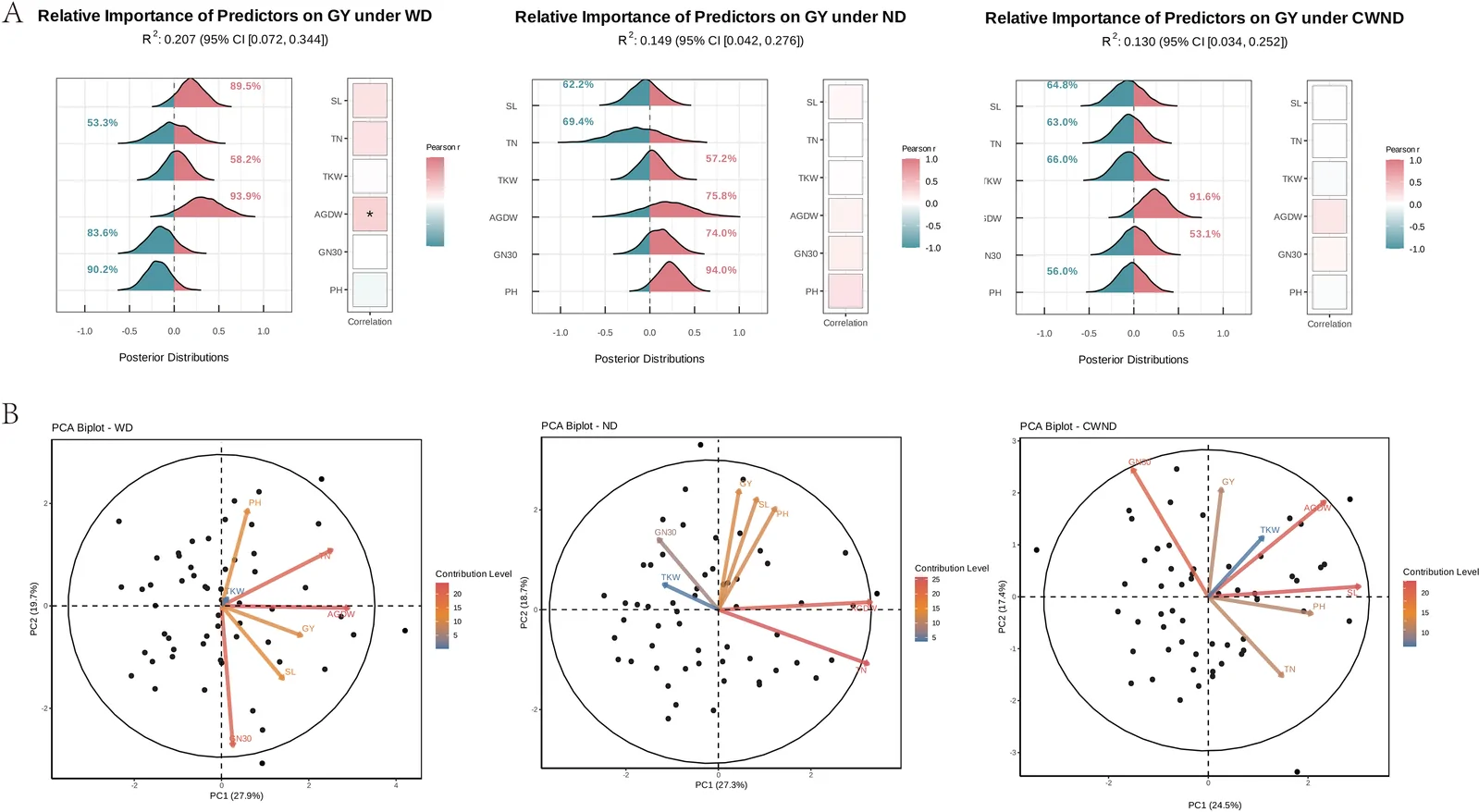
Environment-dependent reconfiguration of trait covariation patterns. **(A)** Bayesian posterior distribution and correlation plot. Left panels show Bayesian posterior distributions of trait effects on grain yield (GY), with negative effects in blue and positive effects in red. The percentage values indicate the probability that the effect is positive (red) or negative (blue), with values >90% considered meaningful. Right panels depict Pearson correlation coefficients between each trait and GY; *p < 0.05, **p < 0.01, ***p < 0.001. AGDW, aboveground dry weight; TN, tiller number; SL, spike length; TKW, thousand-kernel weight; GN30, grain number per 30 panicles; PH, plant height. **(B)** principal component analysis (PCA). PCA biplots illustrate the multivariate structure of phenotypic variation among 56 wheat genotypes under four environmental conditions: fully fertilized and irrigated (FFI), nitrogen deficit (ND), water deficit (WD), and combined water–nitrogen deficit (CWND). Each plot displays PC1 vs. PC2, with trait vectors indicating loadings and black dots representing individual genotypes. Vector color reflects contribution level to the respective principal component.

### 3.3 Temporal dynamics of flag leaf SPAD

To assess physiological responses to nitrogen and water limitations during grain filling, we monitored leaf SPAD, a rapid, non-destructive proxy strongly correlated with chlorophyll content at three developmental stages: stem elongation, heading, and grain filling, across 72 genotypes (56 overlapping with the yield trait analysis and 16 additional genotypes assessed only for SPAD; **Supplementary Table S1**) and four treatments.

SPAD values increased significantly from stem elongation to grain filling in all treatments (P < 0.001), indicating a general trend of greening during reproductive development. However, the magnitude of this increase depended strongly on treatment (**Supplementary Table S4**).

The largest gain occurred under FFI, where SPAD rose by 16.3 units from stem elongation to grain filling stage: from 45.5 to 61.9. In contrast, the smallest increase was observed under CWND (10.9 units; 44.2 to 55.1), reflecting the strongest suppression of greenness development.

At stem elongation, SPAD did not differ significantly among treatments. Notably, plants under water deficit had slightly higher SPAD than those under full fertilization though confidence intervals overlapped. ND and CWND showed the lowest values at this stage.

By heading stage, differences in SPAD became apparent. Plants with adequate nitrogen maintained SPAD values above 50, whereas those under nitrogen deficit (ND and CWND) showed lower greenness, regardless of water availability.

At grain filling, SPAD values under water deficit and nitrogen deficit were similar, indicating comparable effects of the two single stresses on flag leaf greenness (**Figure 4**).

**Figure 4:**
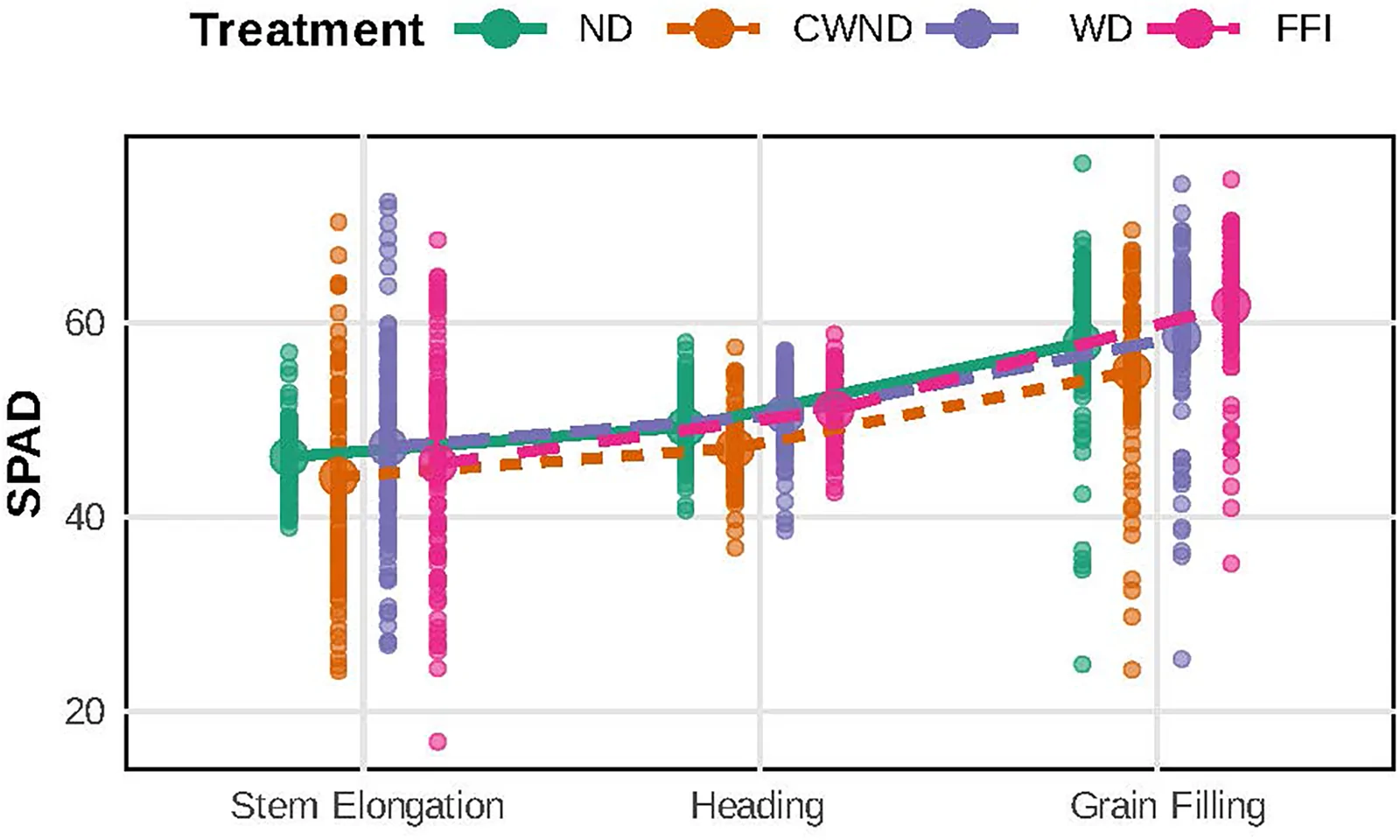
Temporal dynamics of flag leaf SPAD values across nitrogen–water deficit treatments during reproductive development. SPAD readings (a non-destructive proxy for chlorophyll content) were measured at three developmental stages in 72 wheat genotypes under four treatments. Large colored points represent model-based estimated marginal means (EMMs) from the repeated-measures ANOVA; lines connect means within each treatment.

### 3.4 Identifying stress resilient wheat genotypes with high yield

To identify wheat genotypes with high yield stability under nitrogen deficit, we applied a two-dimensional classification based on two key metrics: grain yield under nitrogen deficiency (ND_GY), reflecting absolute performance under nitrogen deficiency condition, and the fertilizer response index (FRI/AYSR), (ND_GY/FFI_GY)/(mean_ND_GY/mean_FFI_GY), defined as the ratio of ND_GY to yield under fully fertilized and irrigated (FFI), normalized by the mean yields across genotypes in each environment, representing relative yield maintenance under stress (Fischer and Wood, 1979). Using median values as thresholds, genotypes were classified into four categories: Dual-Elite, High-Yield Sensitive, Resilient Low-Yield, and Dual-Low. Among them, 10 genotypes were classified as Dual-Elite, representing those that maintain both high productivity and strong yield stability under nitrogen limitation. When genotypes were ranked by grain yield under nitrogen deficit (ND_GY), the top performers included JM47, JM325, TN18, JH927, XM296, DGP8, JM22, H0628, XM28, and HM13-4470, highlighting their high productivity in low-nitrogen environments. In contrast, ranking by fertilizer response index identified LY502, H4399, DGP8, HN1412, SN28, H6172, SN29, TN18, JH927, and XM296 as the most resilient genotypes. Only four genotypes, TN18, JH927, XM296, and DGP8, ranked among the top 10 in both ND_GY and FRI. To further validate this result, we calculated a composite index as the geometric mean of ND_GY and FRI ( $\sqrt{\text{ND}\_\text{GY }\times \text{ FRI}}$). All four overlapping genotypes ranked within the top 10 for the composite index, reinforcing their dual advantage. ​​Consequently, these genotypes are promising for improving crop performance under nitrogen-limited conditions because they performed well in both ​​absolute yield under stress​​ and ​​relative yield maintenance (**Figure 5A**).

**Figure 5:**
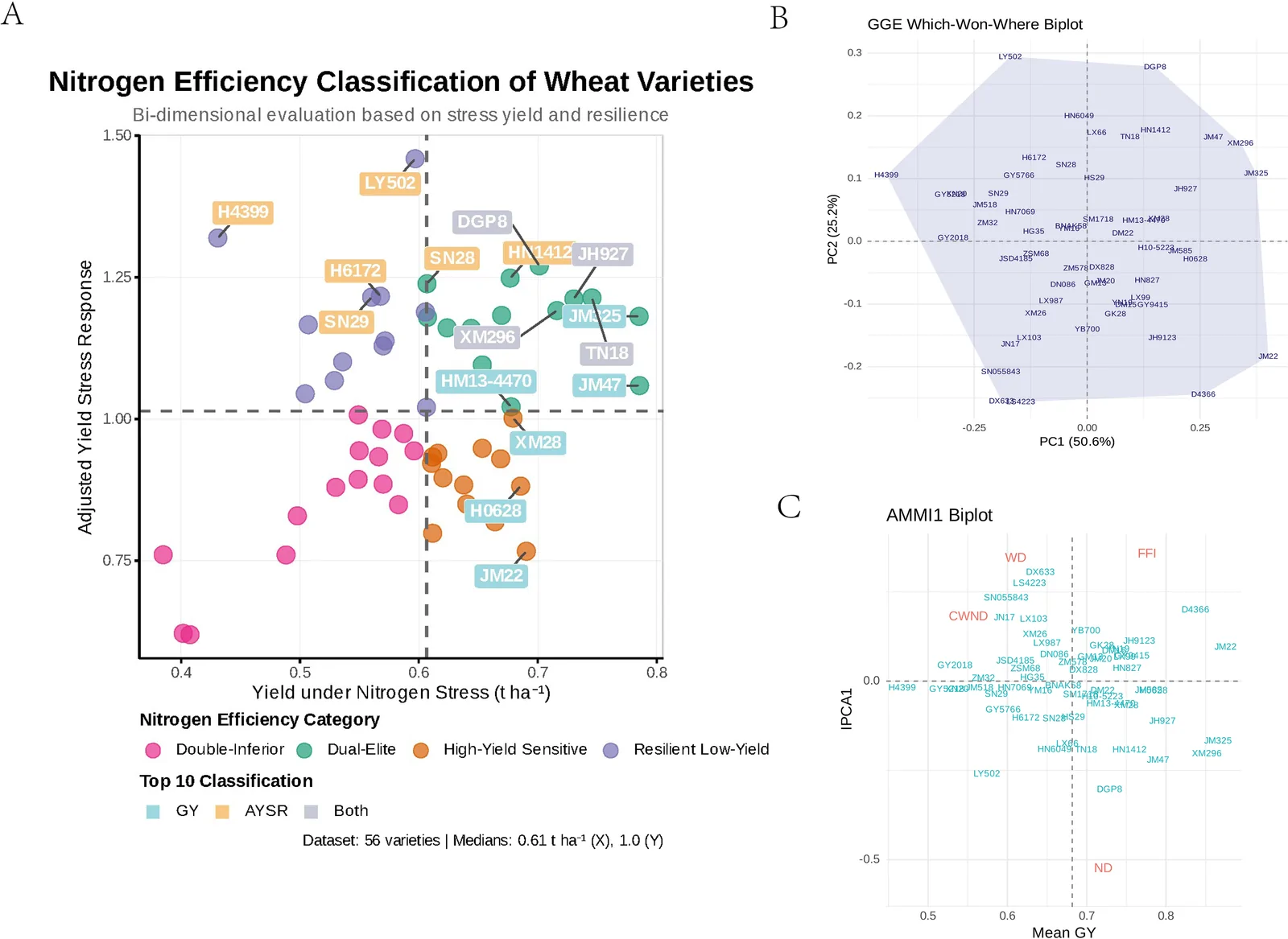
Yield performance classification and genotype-by-environment (G×E) interaction analysis of wheat genotypes across four environments. **(A)** Two-dimensional classification of wheat genotypes for yield performance under nitrogen deficit​ based on yield under nitrogen deficit and resilience to fertilizer reduction. Genotypes were classified into four categories using two key metrics: (1) grain yield under nitrogen deficit (ND_GY, x-axis), reflecting absolute performance in low-nitrogen environments; and (2) adjusted yield stress response (AYSR/FRI), defined as the ratio of ND_GY to FFI_GY normalized by mean yields across genotypes [(ND_GY/FFI_GY)/(mean_ND_GY/mean_FFI_GY)], representing relative yield maintenance under stress (y-axis). **(B)** GGE biplot illustrating the “which-won-where” pattern, mean yield, and stability of genotypes across the four environments (FFI, ND, WD, and CWND). Vertex genotypes indicate specific adaptation to target environments, while genotypes near the origin exhibit broad stability. **(C)** AMMI1 biplot displaying the main effects (mean yield, x-axis) and the first interaction principal component axis (IPCA1, y-axis) for grain yield.

We then applied the same two-axis strategy to two additional stress environments: water deficit (WD) and integrated nitrogen and water deficit (CWND). Under WD, the genotypes that ranked in the top 10 for both high yield (WD_GY) and good yield maintenance (WRI, (WD_GY/FFI_GY)/(mean_WD_GY/mean_FFI_GY)) were XM296, JM325, DX633, and JM20. Under CWND, a different set of genotypes, JM325, JH927, HN1412, and BNAK58 emerged as the top all-rounders. A cross-comparison of these results revealed that no single genotype consistently ranked among the elite across all three stress environments. Notably, XM296 was the only genotype to rank among the elite in both nitrogen-deficient and water-deficit environments, demonstrating this dual excellence under both nitrogen and water deficit (**Supplementary Figure S5**).

To examine the genotype × environment interaction (G×E) for grain yield under the contrasting water–nitrogen regimes, we conducted AMMI and GGE biplot analyses based on plot-level mean yield data. The GGE biplot (**Figure 5B**) explained 75.8% of the total G+GE variation (PC1: 50.6%; PC2: 25.2%). The polygon view identified genotypes at the vertices as the highest-yielding candidates in specific directions. Genotypes such as LY502, JM22, and D4366 were among the vertex genotypes. Across the biplot, genotypes varied in their projection onto PC2: YM16 and DM22 were positioned close to the PC1 axis, indicating relatively stable yield performance across environments, whereas vertex genotypes including LY502 and DX633 showed larger PC2 values, reflecting greater responsiveness to specific conditions. The AMMI1 biplot (**Figure 5C**) displayed the distribution of genotypes and environments along mean yield (x-axis) and the first interaction principal component axis (IPCA1, y-axis). Among the four environments, FFI had the highest mean yield, followed by ND and WD, while CWND had the lowest mean yield. On the IPCA1 axis, ND was separated at the negative end, whereas FFI, WD, and CWND clustered on the positive side, indicating that the genotype yield ranking under ND differed from that under the other three regimes. The environments also differed in discriminatory power: FFI and WD showed larger distances from the origin along IPCA1, suggesting stronger differentiation of genotype rankings.

### 3.5 Comprehensive evaluation of stress resilience using multiple agronomic traits​

To move beyond grain yield alone, we developed a multi-trait evaluation system integrating all seven measured traits across stress environments. This comprehensive evaluation aims to identify genotypes that perform well across all deficit conditions. For each genotype and treatment, trait performance was expressed as a stress response ratio: the value under stress divided by the corresponding value under FFI. This simple ratio helps to factor out innate differences between genotypes, focusing the analysis on their specific response to stress.

We then used principal component analysis (PCA) to combine these seven trait ratios into a smaller set of independent components. We retained components with an eigenvalue greater than 1, which resulted in three principal components for each stress environment. The eigenvalues for the first three PCs under ND, WD, and CWND were (2.16, 1.64, 1.10), (1.98, 1.58, 1.21), and (1.79, 1.38, 1.06), respectively. Together, these three components consistently explained approximately 70% of the total variance in the multi-trait data for each environment, confirming they capture the main patterns of genotypic response. PC1 was characterized by high positive loadings of AGDW and TN across all three stress conditions. Plant height (PH) showed negative loadings on PC1 in the WD/FFI and CWND/FFI analyses, while its loading was positive in ND/FFI. On PC2 and PC3, GN30 and TKW exhibited variable loadings across environments, with both traits often showing positive associations on PC2. Other traits exhibited stress-specific patterns in their association with the principal components. For instance, SL was primarily associated with PC3 under WD, but shifted to PC2 under CWND and to PC1 under ND. Similarly, PH showed a strong positive loading on PC3 only under CWND, while its relationship with PC1 was consistently negative under both WD and ND. The distribution of trait loadings across PC1, PC2, and PC3 varied across stress types, reflecting differences in the structure of phenotypic variation under water deficit, nitrogen deficiency, and their combination (**Supplementary Table S5**).

To construct a comprehensive evaluation index, the scores of each genotype on the first three principal components (PCs) were first transformed using a membership function, normalizing the data to a comparable 0–1 scale. The relative contribution of each PC to the integrated score was then determined through the entropy weight method, which assigns weights objectively based on the information divergence of the membership values themselves. This approach ensures that components with greater variation in genotype performance receive higher weights. The resulting entropy-based weights for each PC under the three stress environments are provided (**Table 2**).

**Table 2:** Component matrix and the cumulative contribution rate of principal components.

Trait	WD PC1	WD PC2	WD PC3	CWND PC1	CWND PC2	CWND PC3	ND PC1	ND PC2	ND PC3
CCR (%)	0.283	0.508	0.681	0.255	0.452	0.603	0.308	0.543	0.701
λ	1.980	1.577	1.210	1.785	1.381	1.056	2.158	1.641	1.105
FW	0.305	0.274	0.421	0.367	0.375	0.258	0.450	0.172	0.378
TKW	0.387	-0.339	0.297	0.152	0.523	-0.198	0.220	0.569	0.294
GN30	0.432	-0.442	0.293	0.388	0.173	-0.312	0.275	0.510	0.260
TN	0.459	0.451	-0.387	0.638	-0.143	0.056	0.495	-0.469	0.109
AGDW	0.576	0.400	-0.037	0.634	-0.226	0.205	0.595	-0.317	0.115
SL	0.067	0.322	0.608	-0.107	-0.485	-0.166	0.383	0.171	0.081
PH	-0.341	0.373	0.196	-0.054	-0.028	0.854	-0.311	-0.183	0.483
GY	-0.032	0.291	0.517	0.054	0.624	0.248	-0.183	-0.181	0.762

CCR, Cumulative Contribution Ratio; FW, Factor Weight.

A final integrated resilience score (D-value) for each genotype was subsequently calculated as the weighted sum of its three membership-transformed PC scores using these objectively determined entropy weights.

### 3.6 Integrated phenotypic profiling for broad-spectrum stress-resilient genotypes

To comprehensively evaluate stress tolerance, we applied three complementary approaches: cluster analysis based on D-values, a threshold-based classification system, and a percentile ranking strategy (**Supplementary Table S6**).

We first examined the distribution of integrated D-values across nitrogen deficit (ND), water deficit (WD), and their combination (CWND). The scores showed moderate variability(WD: 31.3%, CWND: 30.7%, ND: 22.7%), indicating sufficient discrimination among the 56 genotypes. Hierarchical clustering (Euclidean distance, Ward’s D2) revealed three distinct resilience profiles (**Figure 6A**). Cluster 1 included genotypes with consistently high D-values across all environments, such as YB700, GM13, BNAK58, DX633, HS29, LX99, and XN20, reflecting stable performance under diverse stress conditions. These accessions maintained relatively high biomass and yield component stability, suggesting a robust, balanced response. In contrast, Cluster 2 showed intermediate and often environment-specific performance, while Cluster 3 was dominated by genotypes with low to moderate resilience.

**Figure 6:**
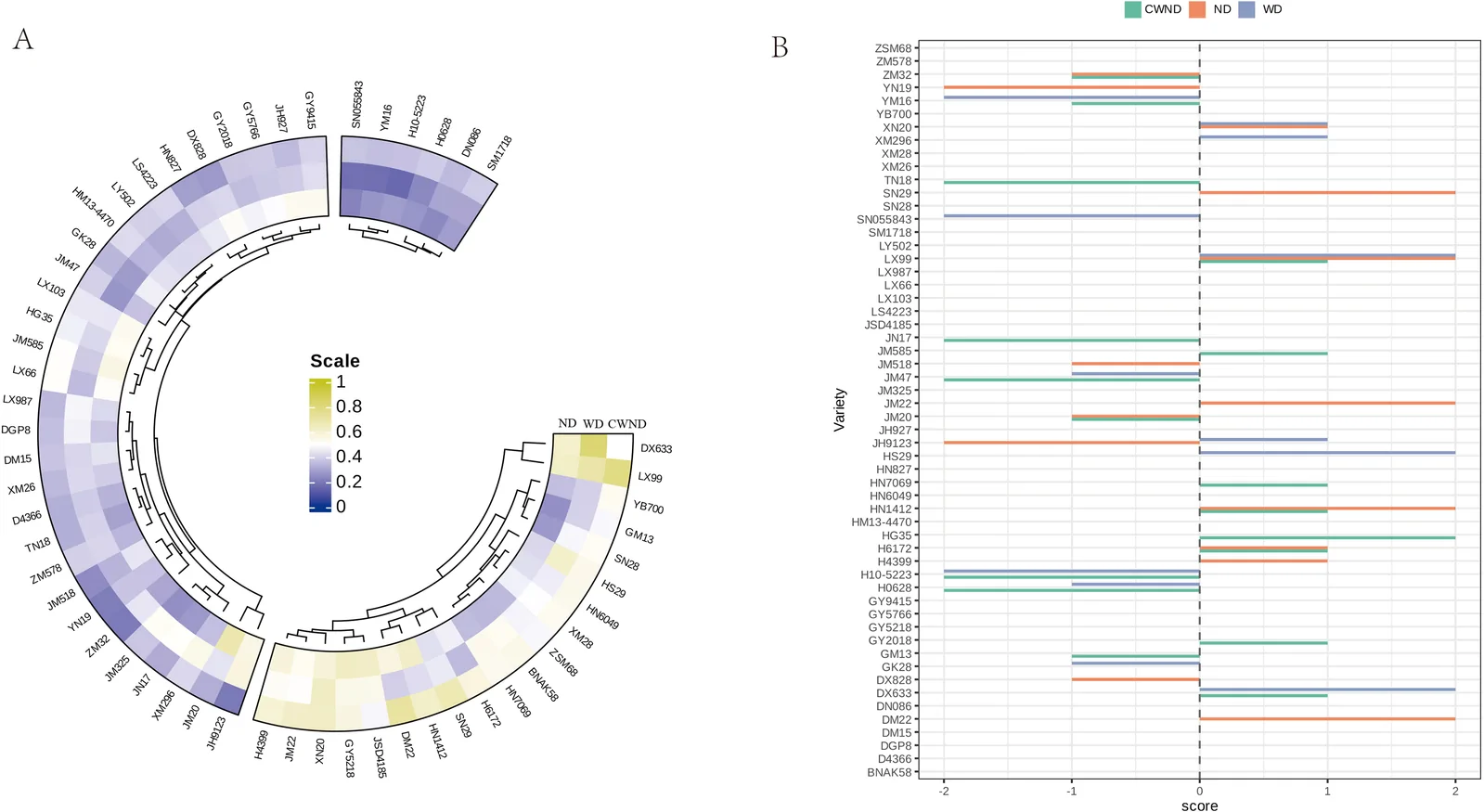
Integrated phenotypic profiling of wheat genotypes under multiple stress environments. **(A)** Circos plot showing hierarchical clustering of 56 wheat genotypes based on their integrated resilience scores (D-values) across nitrogen deficit (ND), water deficit (WD), and combined stress (CWND). Genotypes are grouped into three clusters. The inner ring represents the D-value heatmap, with color intensity reflecting relative resilience (scale: 0–1). The outer arcs show individual D-values for each environment, revealing consistent patterns within clusters. **(B)** Bar plot displaying the numerical classification of genotypes based on D-values converted to a standardized score scale from −2 to +2: Highly tolerant (≥ μ + 1.64σ), Tolerant (μ + 1.00σ to μ + 1.64σ), Moderate (± 1.00σ), Susceptible (μ − 1.64σ to μ − 1.00σ), and Highly susceptible (≤ μ − 1.64σ).

To refine this classification, we applied a threshold-based system using standard deviation cutoffs: Highly tolerant (≥ μ + 1.64σ), Tolerant (μ + 1.00σ to μ + 1.64σ), Moderate (within ±1.00σ), Susceptible (μ − 1.64σ to μ − 1.00σ), and Highly susceptible (≤ μ − 1.64σ). Converting these categories to numerical scores (−2 to +2) allowed direct comparison across environments (**Figure 6B**). The results largely corroborated the clustering: genotypes in Cluster 1, particularly LX99, DX633, and HS29, were classified as highly tolerant or tolerant in all three conditions, reinforcing their status as top performers.

We then shifted from absolute scores to relative ranking, asking which genotypes ranked in the top 30% (≥70th percentile) across all stress environments. Only five genotypes met this criterion: BNAK58, DX633, HS29, LX99, and XN20, all members of Cluster 1. When we extended this ranking to include yield potential under optimal conditions, a clear divergence emerged (**Supplementary Figure S6**): LX99 performed well across all metrics, combining high stress resilience and consistently high​ yield. In contrast, XN20 showed excellent tolerance but ranked near the bottom for yield under non-stress conditions.

## 4 Discussion

In this study, we assessed 56 wheat genotypes across four contrasting environments: optimal conditions (FFI), water deficit (WD), nitrogen deficit (ND), and combined water–nitrogen deficit (CWND) to characterize their performance under key abiotic constraints. Rather than relying on grain yield alone, we integrated multiple agronomic traits into a resilience index based on stress-to-optimal ratios (stress:FFI). This index was derived using principal component analysis (PCA) combined with the entropy weight method, which assigns greater weight to components that exhibit higher inter-genotypic variation. By doing so, we aimed to identify genotypes that combine relatively stable performance under stress with high yield potential under favorable conditions (**Figure 7**).

**Figure 7:**
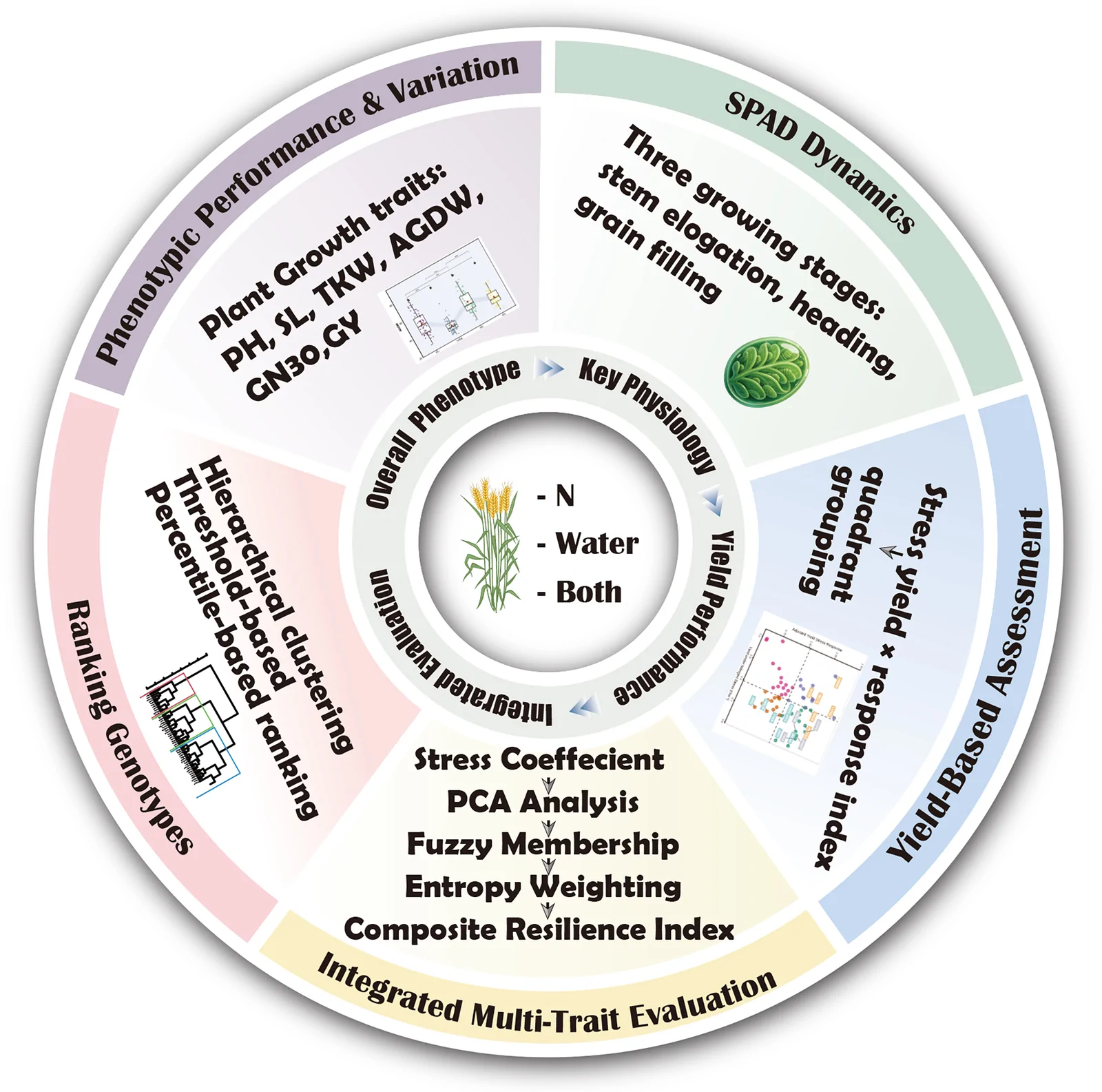
Conceptual framework for integrated evaluation of wheat genotypes under nitrogen and water deficit.

### 4.1 Impact of water and nitrogen deficits on agronomic traits

Grain yield and most associated traits were reduced under both water and nitrogen deficits, though the magnitude and pattern of reduction varied slightly between treatments. Under WD, grain yield averaged 0.61 ± 0.11 kg, representing a 21% decrease relative to FFI (0.77 ± 0.14 kg; p < 0.05). Concurrent declines were observed in aboveground dry weight (–18%) and tiller number (–15%), consistent with previous reports linking water limitation to reduced vegetative growth and biomass accumulation (**Figure 2**) (Garabet et al., 1998; Cui et al., 2015).

Nitrogen deficit also reduced grain yield to a similar extent (0.60 ± 0.09 kg; –22% vs. FFI, p < 0.05), but the largest numerical decline occurred in grain number per 30 spikes (GN30), which dropped from 1091 ± 180 in FFI to 988 ± 158 under ND. This aligns with earlier findings that nitrogen availability during reproductive development strongly influences spike fertility and grain set (Garabet et al., 1998; Zhang et al., 2024). Under combined stress (CWND), grain yield declined further to 0.53 ± 0.08 kg (–31% vs. FFI), and GN30 reached its lowest value (856 ± 128). Tukey’s HSD test confirmed that CWND resulted in significantly lower GY and GN30 than either single stress (p < 0.05), indicating that the combination of water and nitrogen deficits caused a greater reduction in yield than either stress alone.

Trait variability, as measured by the coefficient of variation (CV), was generally highest under FFI. For example, AGDW (FFI 23.4%), and GN30 (FFI 16.5%), suggesting that genetic differences are more fully expressed under non-limiting conditions. Tiller number showed comparable variability between ND (22.4%) and FFI (20.4%), indicating that genotypic divergence in tillering capacity may be maintained even under nitrogen-limited conditions.

To explore how trait associations shift across environments, we conducted separate PCA for each condition. The first two principal components accounted for 46–47% of total variation in all cases, indicating comparable overall dimensionality. However, the contribution of individual traits to each component varied. In FFI, PC1 was primarily associated with AGDW and TN, whereas in CWND, SL and PH contributed more strongly to PC1, while GY and GN30 loaded more heavily on PC2. This reorganization of trait loadings suggests that the structure of phenotypic covariation is environment-dependent, although the physiological basis for these shifts remains to be clarified (Tanner et al., 2022).

### 4.2 SPAD dynamics highlight the dominant role of nitrogen in chlorophyll maintenance

The measurement of flag leaf SPAD values was conducted at three critical growth stages, stem elongation, heading, and grain filling to capture the dynamics of chlorophyll content across the transition from vegetative growth to reproductive development and most importantly, through the grain filling period, which is paramount for yield determination. Flag leaf SPAD increased from stem elongation to early grain filling in all treatments, which is consistent with the plant’s strategy to maximize photosynthetic capacity just as grain development begins. The flag leaf supplies most of the carbohydrates used to fill the grain, so maintaining its greenness during this period is considered beneficial for high yield (**Figure 4**) (Kong et al., 2010; Vicente et al., 2018; Croce et al., 2024).

Our findings that flag leaf SPAD values increased significantly from stem elongation to grain filling across all treatments (P < 0.001) are consistent with active chlorophyll synthesis during reproductive development. This pattern is consistent with the view that the photosynthetic capacity of the flag leaf is an important contributor to grain yield. This is supported by previous research. Study confirmed that rice yield was significantly correlated with both photosynthetic rate and SPAD value of the flag leaf at 30 days after heading, directly demonstrating the critical importance of the flag leaf’s physiological state post-heading (Liu et al., 2020). The magnitude of increase, however, differed among treatments: FFI showed the largest gain, while CWND showed the smallest. Notably, at the heading stage, genotypes grown under nitrogen-deficient conditions (ND and CWND) consistently maintained SPAD values below 50, regardless of water status, whereas those with adequate nitrogen exceeded 50. This pattern supports the view that nitrogen availability is the primary factor governing chlorophyll retention during grain filling, with water playing a secondary role (Gholizadeh et al., 2017). By early grain filling, SPAD values under WD and ND converged, suggesting comparable rates of senescence under single stresses. The fact that genotypes such as XM296 maintained relatively high SPAD (>58) under both ND and WD, which corresponded with their higher grain yields reinforces the stay-green trait, as a potentially useful indicator of stress resilience, given its association with sustained greenness during the critical grain-filling period.

### 4.3 From broad adaptation toward targeted deployment: a potalaization model for wheat

The pronounced genotype-by-environment interactions observed in this study highlight that stress resilience in wheat is highly dependent on both the evaluation framework and environmental context. Indeed, grain yield itself is a complex, polygenic trait that is particularly vulnerable to genotype-by-environment interactions under abiotic stress, often resulting in low heritability (Golabadi et al., 2005; Maniee et al., 2009). This helps explain why selection strategies based solely on grain yield—whether absolute stress yield or normalized stress:FFI ratios—tend to lack stability across heterogeneous environments.

The AMMI1 described the distribution of the four environments along mean yield (x-axis) and the first interaction principal component axis (IPCA1, y-axis). ND was positioned at the negative end, whereas FFI, WD and CWND were located on the positive side, indicating that the yield ranking pattern under nitrogen deficit without water stress differed from that under water-related regimes. The relatively close positions of WD and CWND on IPCA1 suggested that water deficit contributed prominently to the interaction pattern under combined stress. This is consistent with winter-wheat studies showing that water supply restricts nitrogen uptake and yield formation, and that severe water deficit can reduce the responsiveness to nitrogen application (Zhuang et al., 2024). In broader wheat G×E research, water deficit and related abiotic constraints have been reported to generate substantial genotype-by-environment interactions (Parent et al., 2017). In the North China Plain, water shortage during the winter wheat growing season, especially during spring pre-anthesis and late spring reproductive periods, is widely reported as a major constraint on grain yield (Wu et al., 2014; Zeng et al., 2023). Wheat responses to nitrogen application are further regulated by soil moisture availability and irrigation regime. These results indicate that yield-based rankings were environment-dependent and varied according to the type of stress, which supports the use of a multi-trait framework rather than grain yield alone.

Within the yield-centric evaluation system, genotypes such as XM296, TN18, and JH927 were identified as “high-stability” candidates due to their relatively high stress:FFI yield ratios. However, this assessment reflects performance in only one trait and does not capture responses in other agronomic or physiological dimensions. In contrast, the multi-trait D-value framework revealed a distinct set of broadly resilient genotypes: BNAK58, DX633, HS29, LX99, and XN20—all of which maintained D-values in the top 30% across nitrogen deficit (ND), water deficit (WD), and combined water–nitrogen deficit (CWND) environments, placing them in Cluster 1. Notably, LX99 combines this broad resilience with high yield potential (ranking in the top 30% under optimal conditions), whereas XN20, despite similarly high D-values, ranks in the bottom 20% for FFI yield, underscoring their divergent breeding values (**Figure 5**, **6**).

XM296 was not part of this core resilient group; instead, it was assigned to Cluster 2, indicating inconsistent multi-trait responses across stresses. Particularly under combined stress (CWND), traits such as biomass and tiller number declined markedly, limiting its overall resilience score. In other words, XM296’s apparent advantage is confined to the narrow metric of yield ratio and does not reflect comprehensive stress tolerance. These findings support a shift from the traditional paradigm of “broadly adapted mega-varieties” toward a targeted “Potalaization” model of wheat improvement (Wu et al., 2023), a concept originally proposed by Wu et al. referring to targeted deployment of genotypes to specific production environments. Genotypes like XM296 are best suited for regions dominated by predictable, single stresses (e.g., rainfed low-nitrogen systems), where yield-ratio performance is prioritized; LX99 represents a favorable balance, offering both high baseline yield and multi-trait stability across fluctuating or compound stresses, positioning it as a candidate of interest for production zones facing increasing climate uncertainty.

It is crucial to emphasize that the D-value framework is not intended to supersede grain yield as the ultimate breeding objective. Instead, it serves as a complementary tool to navigate the challenges posed by high G×E. By capturing a genotype’s capacity to maintain core agronomic functions across diverse stress scenarios, the D-value helps identify lines with a higher probability of delivering reliable, non-catastrophic yields in variable environments. In an era of increasing climatic volatility, such stability is a critical component of long-term productivity and food security. It should be noted that composite indices constructed through multi-step transformations inevitably involve a degree of information condensation, and the entropy weighting procedure employed here places greater emphasis on the discriminative capacity of each principal component rather than its variance contribution. While this distinction reflects a deliberate methodological choice, the biological interpretation of the resulting D-value ultimately depends on the quality and relevance of the input traits. Taken together, these observations offer a preliminary indication that the D-value framework may serve as a useful complement to yield-based evaluations in identifying genotypes with coordinated multi-trait performance under stress. Further refinement of the weighting strategy and inclusion of additional physiological or molecular indicators may enhance the resolution of this framework in future applications.

## Data Availability

The original contributions presented in the study are included in the article/**Supplementary Material**. Further inquiries can be directed to the corresponding author/s.

## Supplementary material

Supplementary Figure 1 Trait-specific radar plots showing phenotypic performance of 56 wheat genotypes across four environmental treatments (FFI, WD, ND, CWND).

Supplementary Figure 2 Boxplots of distribution patterns for seven key agronomic traits across the four treatments.

Supplementary Figure 3 Distribution and statistical comparison of key agronomic traits across nitrogen–water deficit treatments.

Supplementary Figure 4 Pearson correlation coefficients among agronomic traits.

Supplementary Figure 5 Water Efficiency Classification of Wheat Varieties

Supplementary Figure 6 Integrated D-values and yield potential under optimal conditions for top-ranked genotypes.

Supplementary Table 1 Genotypic information for 72 wheat accessions

Supplementary Table 2 Coefficient of variation, skewness, and kurtosis for agronomic traits under stress.

Supplementary Table 3 Pearson correlation coefficients among agronomic traits.

Supplementary Table 4 Tukey-adjusted pairwise comparisons of flag leaf SPAD values across nitrogen–water treatments and developmental periods in wheat.

Supplementary Table 5 PCA component matrix for multi-trait stress response ratios.

Supplementary Table 6 Classification of wheat genotypes based on integrated resilience scores (D-values).
