# Supplementary material for: Phenotypic characterization and resilience assessment of wheat genotypes under single and combined abiotic stresses
Source: Front Plant Sci. 2026 Oct 15;17:1954462. doi: 10.3389/fpls.2026.1954462 (PMC13716657; doi:10.3389/fpls.2026.1954462)
Supplement: Supplementary file 1 [file DataSheet1.pdf]

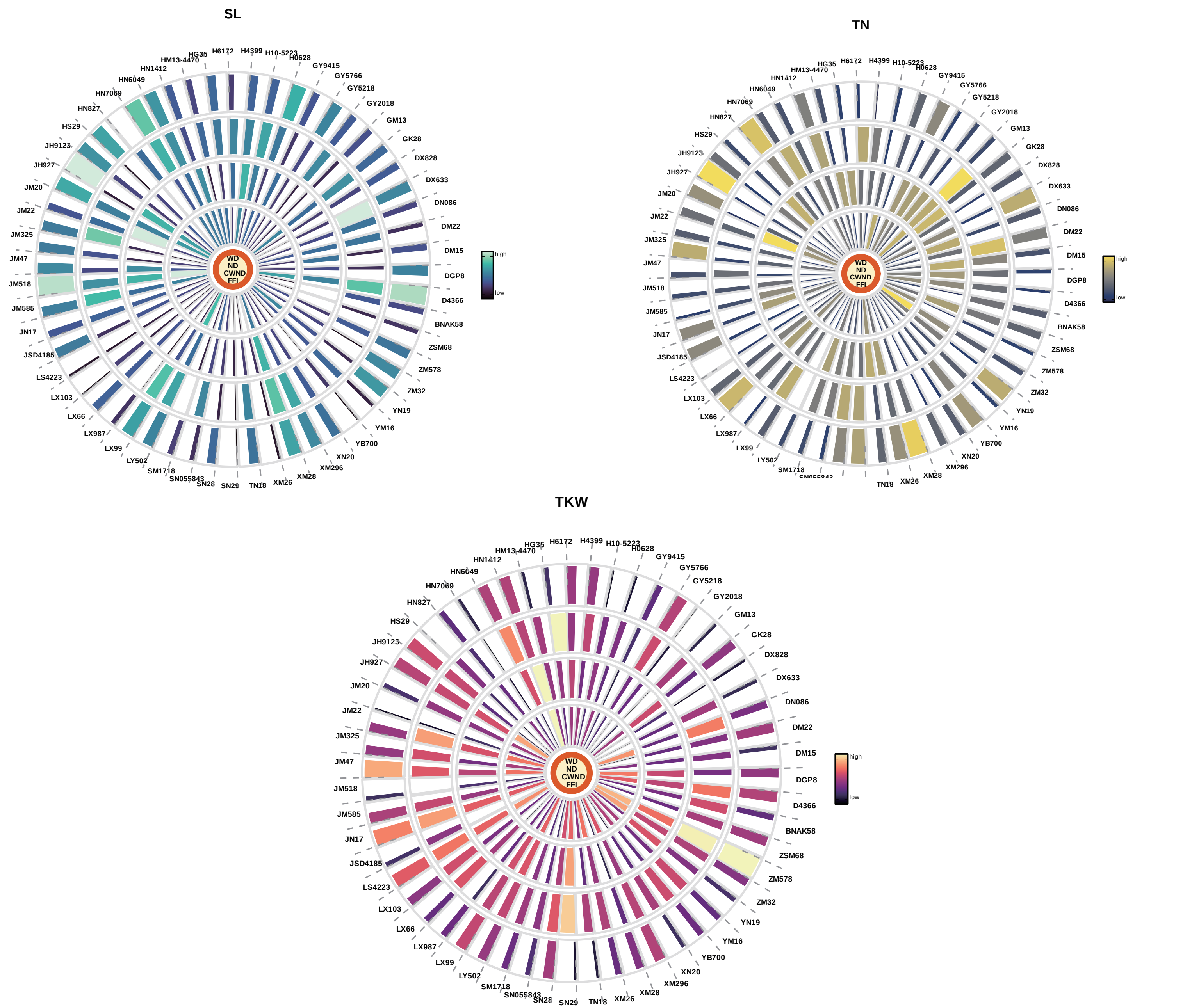
SL
H6172
H4399
H10-5223
H0628
GY9415
GY5766
GY5218
GY2018
GM13
GK28
DX828
DX633
DN086
DM22
DM15
DGP8
D4366
BNAK58
ZSM68
ZM578
ZM32
YN19
YM16
YB700
XN20
XM296
XM28
XM26
TN18
SN29
SN28
SN055843
SM1718
LY502
LX99
LX987
LX66
LX103
LS4223
JSD4185
JN17
JM585
JM518
JM47
JM325
JM22
JM20
JH927
JH9123
HS29
HN827
HN7069
HN6049
HN1412
HM13-4470
HG35
WD
ND
CWND
FFI
high
low
TN
WD
ND
CWND
FFI
high
low
TKW
WD
ND
CWND
FFI
high
low
