# Supplementary material for: Phenotypic characterization and resilience assessment of wheat genotypes under single and combined abiotic stresses
Source: Front Plant Sci. 2026 Oct 15;17:1954462. doi: 10.3389/fpls.2026.1954462 (PMC13716657; doi:10.3389/fpls.2026.1954462)
Supplement: Supplementary file 2 [file DataSheet2.pdf]

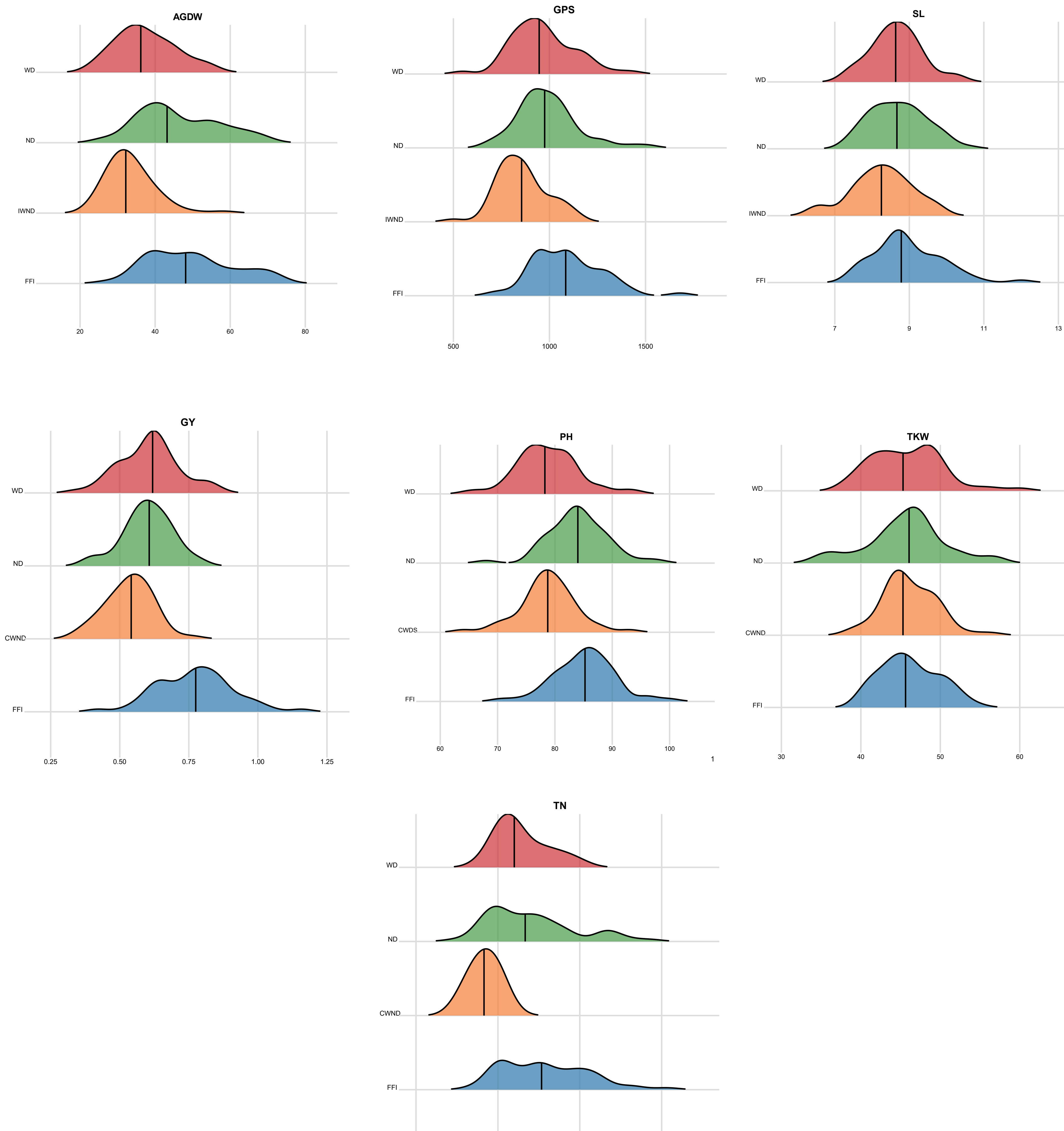

Phenotypic distribution patterns of major agronomic traits across different environmental conditions.

Ridge plots display the normalized trait distributions of 56 wheat genotypes under four treatments well-fertilized and irrigated (FFI, blue), water deficit (WS, red), nitrogen deficit (NS, green), and combined water − nitrogen deficit (IWNS, orange). Traits include aboveground dry weight (AGDW), grain number per spike (GPS), grain yield (GY), plant height (PH), spike length (SL), thousand-kernel weight (TKW), and tiller number (TN). Each column represents a trait; each row corresponds to a treatment. Vertical lines indicate mean values across genotypes within each environment. The broader and higher peaks in FFI suggest greater phenotypic variation and performance, while narrower and lower distributions under IWNS reflect reduced expression and increased selection pressure under combined stress.
