# Supplementary material for: Phenotypic characterization and resilience assessment of wheat genotypes under single and combined abiotic stresses
Source: Front Plant Sci. 2026 Oct 15;17:1954462. doi: 10.3389/fpls.2026.1954462 (PMC13716657; doi:10.3389/fpls.2026.1954462)
Supplement: Supplementary file 3 [file DataSheet3.pdf]

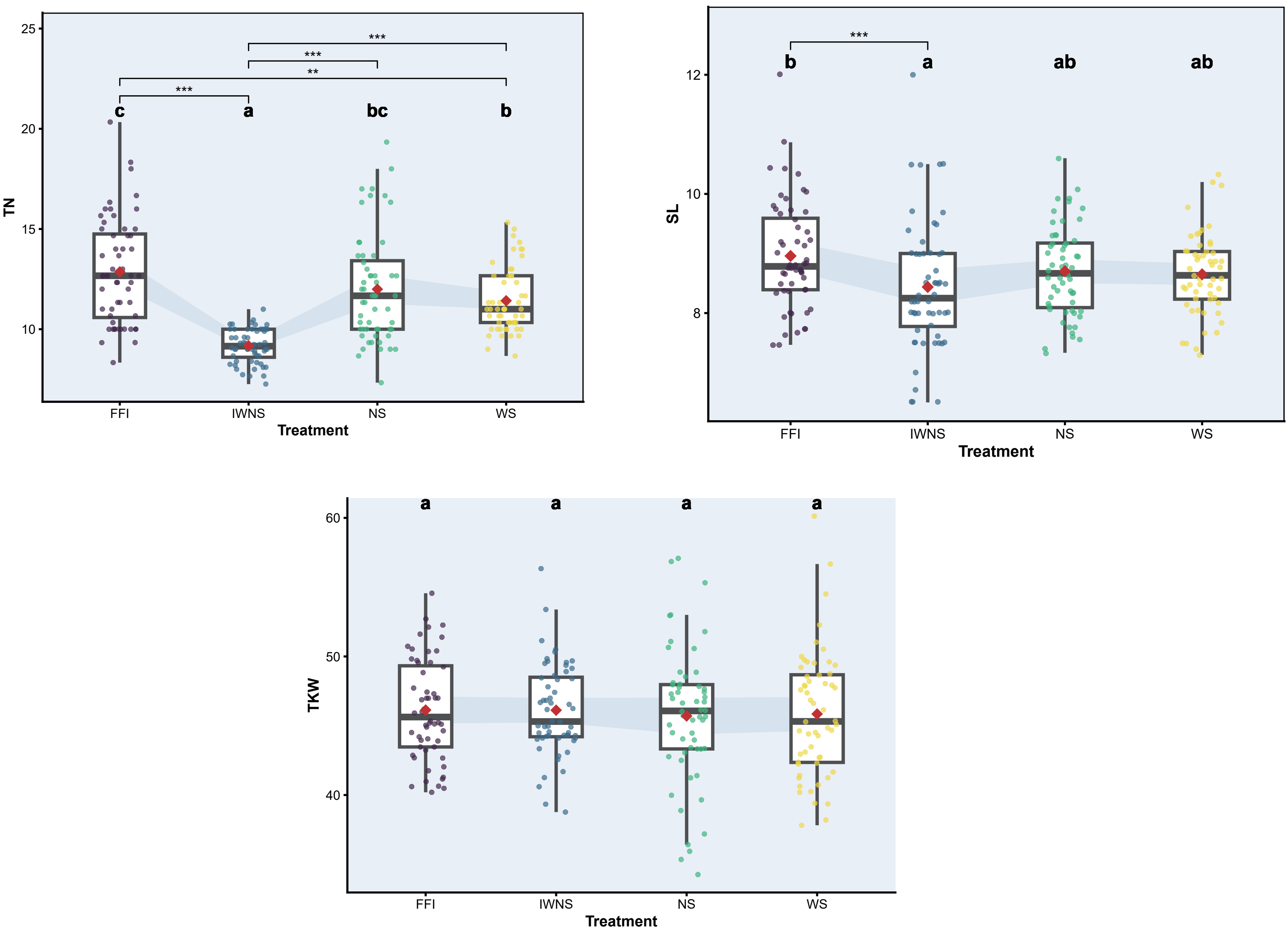

TN
25
20
15
10
***
***
**
***
c
a
bc
b
FFI
IWNS
NS
WS
Treatment
SL
12
10
8
***
b
a
ab
ab
FFI
IWNS
NS
WS
Treatment
TKW
60
50
40
a
a
a
a
FFI
IWNS
NS
WS
Treatment
