# Supplementary material for: Phenotypic characterization and resilience assessment of wheat genotypes under single and combined abiotic stresses
Source: Front Plant Sci. 2026 Oct 15;17:1954462. doi: 10.3389/fpls.2026.1954462 (PMC13716657; doi:10.3389/fpls.2026.1954462)
Supplement: Supplementary file 4 [file DataSheet4.pdf]

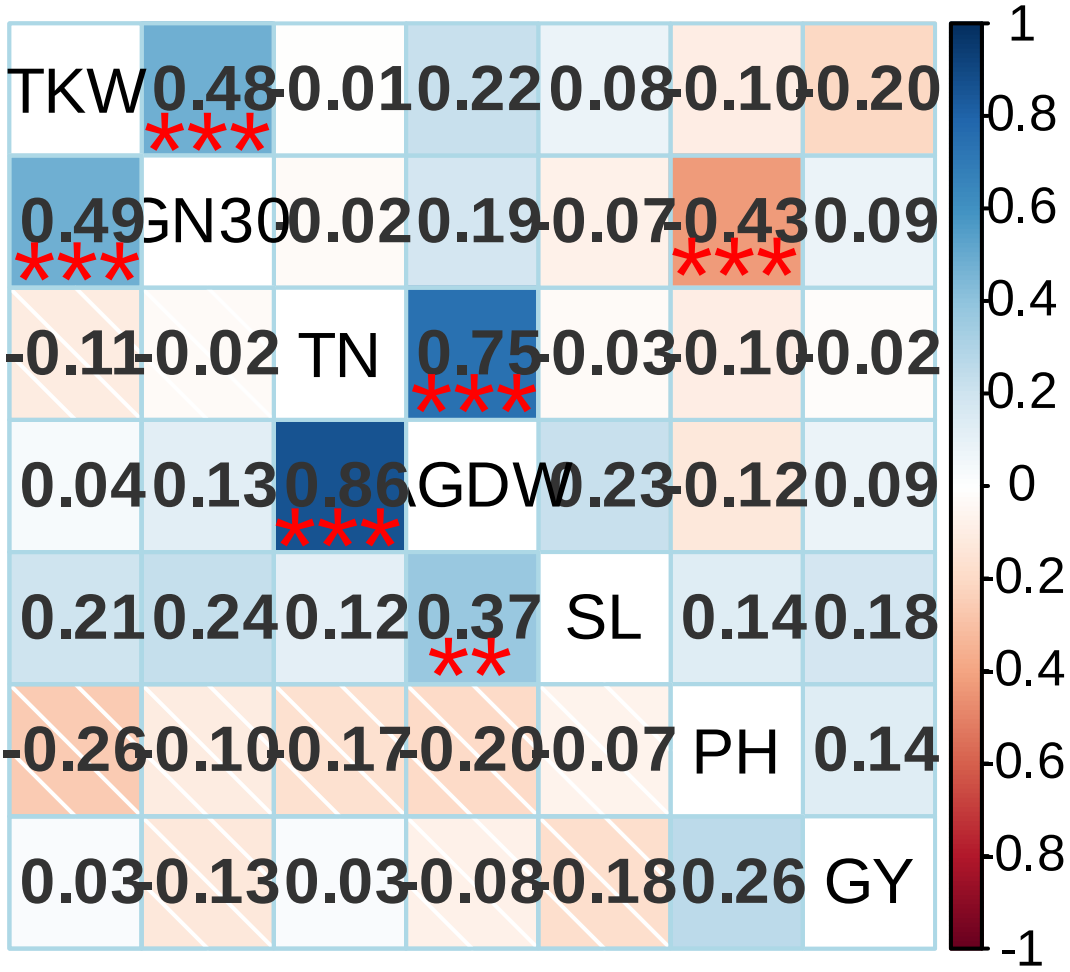

TKW
GN30
TN
GDW
SL
PH
GY
0.48 ***
-0.01
0.22
0.08
-0.10
-0.20
0.49 ***
-0.02
0.19
-0.07
-0.43 ***
0.09
-0.11
-0.02
0.75 ***
-0.03
-0.10
-0.02
0.04
0.13
0.86 ***
0.23
-0.12
0.09
0.21
0.24
0.12
0.37 **
0.14
0.18
-0.26
-0.10
-0.17
-0.20
-0.07
0.14
0.03
-0.13
0.03
-0.08
-0.18
0.26
1
0.8
0.6
0.4
0.2
0
-0.2
-0.4
-0.6
-0.8
-1
