# Supplementary material for: Phenotypic characterization and resilience assessment of wheat genotypes under single and combined abiotic stresses
Source: Front Plant Sci. 2026 Oct 15;17:1954462. doi: 10.3389/fpls.2026.1954462 (PMC13716657; doi:10.3389/fpls.2026.1954462)
Supplement: Supplementary file 5 [file DataSheet5.pdf]

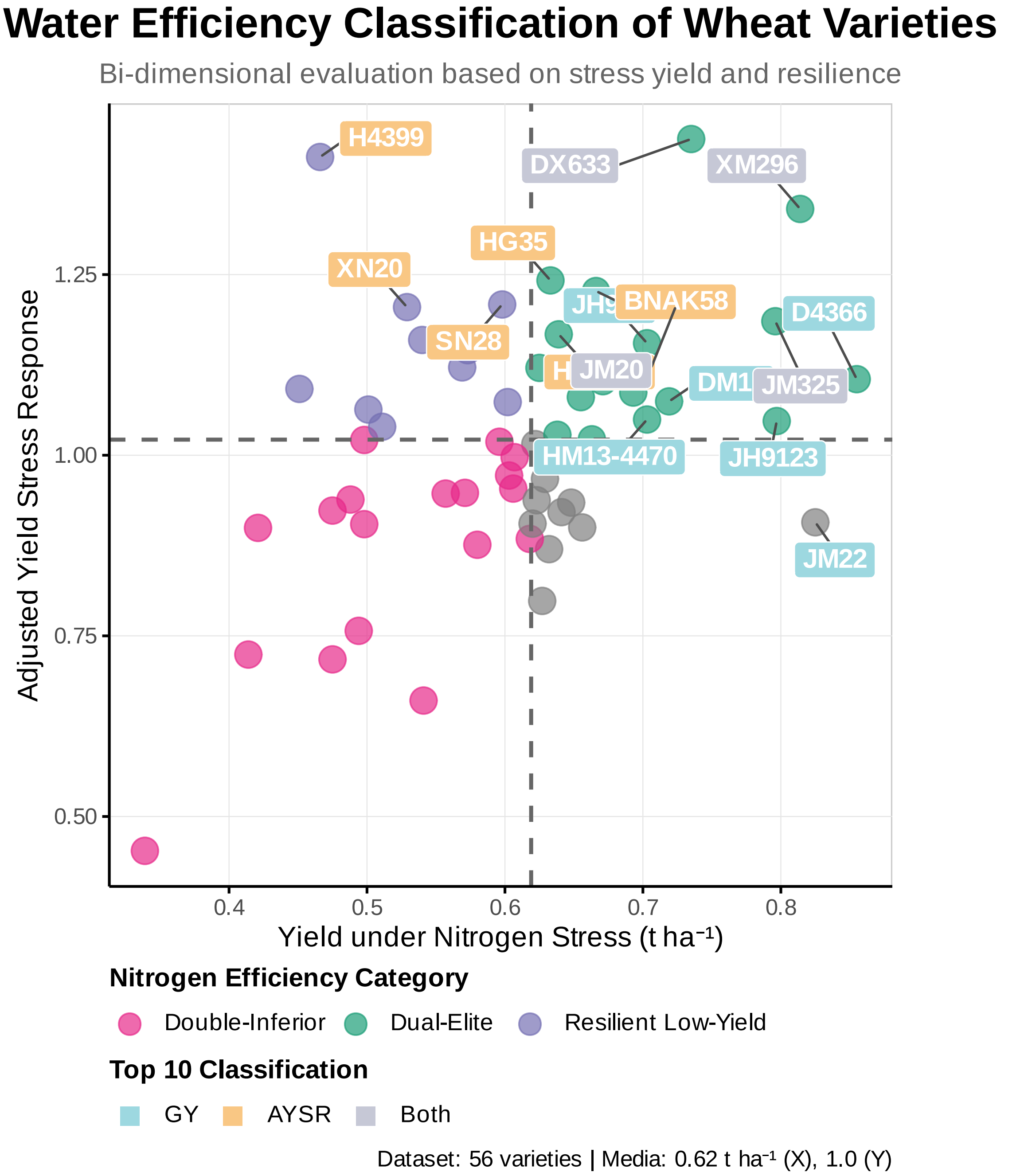

Water Efficiency Classification of Wheat Varieties
Bi-dimensional evaluation based on stress yield and resilience
Adjusted Yield Stress Response
Yield under Nitrogen Stress (t ha⁻¹)
0.4
0.5
0.6
0.7
0.8
0.50
0.75
1.00
1.25
H4399
DX633
XM296
HG35
XN20
BNAK58
D4366
SN28
JM20
DM1
JM325
HM13-4470
JH9123
JM22
Nitrogen Efficiency Category
Double-Inferior
Dual-Elite
Resilient Low-Yield
Top 10 Classification
GY
AYSR
Both
Dataset: 56 varieties | Media: 0.62 t ha⁻¹ (X), 1.0 (Y)
