# Supplementary material for: Phenotypic characterization and resilience assessment of wheat genotypes under single and combined abiotic stresses
Source: Front Plant Sci. 2026 Oct 15;17:1954462. doi: 10.3389/fpls.2026.1954462 (PMC13716657; doi:10.3389/fpls.2026.1954462)
Supplement: Supplementary file 6 [file Table4.docx]

**Tukey-adjusted pairwise comparisons of flag leaf SPAD values across nitrogen–water treatments and developmental periods in wheat**

**Pairwise comparisons of flag leaf SPAD values among nitrogen and water treatments within each developmental period (Tukey-adjusted, α = 0.05)**

**Pairwise comparisons of flag leaf SPAD values across developmental periods within each treatment (Tukey-adjusted, α = 0.05)**
